# Genetic Diversity and Population Differentiation of *Guignardia mangiferae* from “Tahiti” Acid Lime

**DOI:** 10.1100/2012/125654

**Published:** 2012-04-19

**Authors:** Ester Wickert, Eliana Gertrudes de Macedo Lemos, Luciano Takeshi Kishi, Andressa de Souza, Antonio de Goes

**Affiliations:** ^1^Empresa de Pesquisa Agropecuária e Extensão Rural de Santa Catarina (EPAGRI), Estação Experimental de Itajaí, Rodovia Antônio Heil 8400, Itaipava, Itajaí 88318-112, SC, Brazil; ^2^Departamento de Tecnologia da Universidade Estadual Paulista, Faculdade de Ciências Agrárias e Veterinárias de Jaboticabal, Via de Acesso Professor Dr. Paulo Donato Castellane s/n, Jaboticabal 14884900, SP, Brazil; ^3^Departamento de Fitossanidade da Universidade Estadual Paulista, Faculdade de Ciências Agrárias e Veterinárias de Jaboticabal, Via de Acesso Prof. Dr. Paulo Donato Castellane s/n, Jaboticabal 14884900, SP, Brazil

## Abstract

Among the citrus plants, “Tahiti” acid lime is known as a host of *G. mangiferae* fungi. This species is considered endophytic for citrus plants and is easily isolated from asymptomatic fruits and leaves. *G. mangiferae* is genetically related and sometimes confused with *G. citricarpa* which causes Citrus Black Spot (CBS). “Tahiti” acid lime is one of the few species that means to be resistant to this disease because it does not present symptoms. Despite the fact that it is commonly found in citric plants, little is known about the populations of *G. mangiferae* associated with these plants. Hence, the objective of this work was to gain insights about the genetic diversity of the *G. mangiferae* populations that colonize “Tahiti” acid limes by sequencing cistron ITS1-5.8S-ITS2. It was verified that “Tahiti” acid lime plants are hosts of *G. mangiferae* and also of *G. citricarpa*, without presenting symptoms of CBS. Populations of *G. mangiferae* present low-to-moderate genetic diversity and show little-to-moderate levels of population differentiation. As gene flow was detected among the studied populations and they share haplotypes, it is possible that all populations, from citrus plants and also from the other known hosts of this fungus, belong to one great panmictic population.

## 1. Introduction

 The genus *Guignardia* (kingdom Fungi, phylum Ascomycota, class Dothideomycetes, order Botryosphaeriales, family Botryosphaeriaceae) encompasses around 330 known species, but some of them with an unknown anamorphic phase [1]. Many species considered plant endophytic fungi are classified in this family and genus, and among them, there are *G. mangiferae* and also the causal agents of CBS, *G. citricarpa*, and of fruit rot in guava (*Psidium guajava* L.), *G. psidii*.

 Despite causing foliar and fruit spots in mango (*Mangifera indica*) and guava, *G. mangiferae* was isolated from a wide range of different hosts and was considered endophytic because of the symptomless tissues from which it was isolated. Its hosts include Brazilian tropical plants, such as *Apidosperma polineuron*, *Anacardium giganteum*, *Myracrodroun urundeuva, Spondias mombin*, *Bowdichia nítida* and *Cassia occidentalis* [2]. Citrus plants are also known as hosts of *G. mangiferae* [3–5] and it is considered endophytic to this plant because no symptoms are known to be caused by this fungus in citrus hosts. Isolates obtained by the authors were identified by DNA sequencing the ITS rDNA (ITS1-5.8S-ITS2).

 For phylogenetic analysis among species or higher taxonomic levels, the most common genes for sequencing and comparison reside in the ribosomal RNA (rRNA) gene cluster, including the internal transcribed spacer (ITS) regions ITS1 and 2, the intergenic spacer IGS, 5.8S rRNA, 18S rRNA, and 26S rRNA genes. This is due to the fact that these multicopy genes are highly conserved within a species but can be quite variable among species. Other commonly used genes include the mitochondrial ATPase subunits, beta tubulin, and elongation factor [6].

Studies about phylogeny and molecular systematic of fungi have utilized ITS rDNA because of the higher number of random copies of this sequence dispersed throughout the genome and their uniformity, which is generally maintained by stabilizing selection [15]. Generally, conserved regions that encompass genes 18S and 28S can be used to differentiate individuals at the genus and species levels [7], whereas spacer regions ITS and IGS, which accumulate higher levels of genetic variation, are utilized for studies of species, populations, subpopulations, and even same species individual discrimination [8–10].

 Ideally, the best way to quantify genetic variation in natural populations should be by the comparison of DNA sequences [11]. However, although the methodology for DNA sequencing has been available since 1977, until 2000 the use of DNA sequence data had had little impact on population genetics [12]. These authors reflect that the effort (in terms of both money and time) required to obtain DNA sequence data from a relatively large number of alleles was too substantial.

The introduction of the polymerase chain reaction (PCR), which allows direct sequencing of PCR products and avoids, therefore, their cloning, has changed the situation. Undoubtedly, this has produced a revolutionary change in population genetics. Although population studies at the DNA sequence level are still scarce and primarily carried out in *Drosophila* at present, they will certainly increase in the future [12].

 Considering that Brazil has larger production areas of *G. mangiferae* known hosts as Tahiti acid lime, mango, and guava (*Psidium guajava* L.), it would be helpful to know and understand these fungi population structure. Despite Tahiti acid lime did not show any disease symptom caused by *G. mangiferae*, it could be inoculum source for susceptible cultures as mango, and guava. If we consider that citrus, mango and guava plantations are frequently neighbors, *G. mangiferae* can spread rapidly from one culture to another. So, this disease needs careful observations and monitoring and the knowledge about its population genetic structure would be helpful.

Therefore, to obtain valuable information about the genetic structure of *G. mangiferae *populations, we used the SNP markers found in the ITS1-5.8S-ITS2 region. The objectives of this study were to characterize the population genetics of *G. mangiferae *from different geographic regions and determine the genetic diversity and population differentiation.

## 2. Material and Methods

### 2.1. Sampling

The sampling was done in two different geographic areas: in Estiva Gerbi (Coordinate 22° 16′ 15′′ S, 46° 56′ 42′′ W), São Paulo State, and in Itaboraí district (Coordinate 22° 44′ 51′′ S, 42° 51′ 21′′ W), Rio de Janeiro State. In each place, leaves were collected from 24 different acid lime plants in order to obtain one isolate per plant. In the same places, 40 leaves were collected from one plant in order to obtain 24 isolates from the same plant. This sampling, 40 leaves in a same plant, was also done for three different plants in each geographic place.

### 2.2. Culture Characterization of *Guignardia* sp. in Oatmeal (OA) Media

All *Guignardia* isolates from this study were characterized in oatmeal medium according to Baldassari et al. [13].

### 2.3. Amplification and Sequencing of ITS1-5.8S-ITS2

DNA from isolates was extracted according the Kuramae-Izioka [14] protocol. Amplification of ITS1-5.8S-ITS2 was performed using the primers ITS1/ITS4 [15]. PCR reactions were carried out using 2 *μ*L of buffer 1X (KCl 50 mM, TRIS-HCl 200 mM pH 8.4); 0.8 *μ*L of MgCl_2_ 5 mM; 0.4 *μ*L of each dNTP 10 mM; 0.3 *μ*L Taq DNA polymerase; 5 pmol of each primer, with 60 ng of genomic DNA and sterile water q.s.p. to 20 *μ*L. DNA was amplified in a Termocycler PTC-100 Programmable Thermal Controller MJ Research, Inc., with 1 initial cycle at 94°C during 2 min, 39 cycles at (94°C during 1 min, 1 min at 60°C, and 1 min and 30 sec at 72°C), and 1 final cycle at 72°C for 5 min. Amplified samples were submitted to electrophoresis in agarose 1.2%, containing ethidium bromide (0.5 *μ*g/mL) and 1 Kb DNA Ladder. The samples were observed under UV light with a GEL DOC 1000-BioRad (data not shown). The DNA fragments obtained were purified and submitted to sequencing PCR with a DYEnamic ET Dye Terminator Kit (GE Healthcare) according the manufacturer's instructions. The termocycler conditions were the same as those previous described. DNA fragments were precipitated with isopropanol 75%, washed with ethanol 70%, and resuspended with 3 *μ*L of “loading buffer” (5 : 1 formamide/50 mM EDTA, pH 8.0), heated to a 95°C for 2 min, and then applied to the sequencing gel. Electrophoresis was done in a sequencer *ABI Prism 3700 DNA Sequencer* (Applied Biosytems, Foster City, USA). The ITS region of each isolate was submitted to sequencing two times in the two strand ends (Primer Forward + Primer Reverse).

### 2.4. Analysis of Obtained DNA Sequences

The eletropherograms were obtained using the software* ABI Analysis Data Collection* and converted in nucleotide sequences with* DNA Sequencing Analysis Software *Version 3.3. The DNA sequences were then submitted to softwares Phred/Phrap/Consed [16] and Sequencher (version 4.05 (Gene Codes Corp, Ann Arbor, USA)), in order to verify quality and to perform alignment and edition. All the DNA sequences obtained were submitted to GenBank-NCBI for comparison with the deposited sequences using the tool BLAST [17].

### 2.5. Intra- and Intergroup Genetic Distances

Genetic distances were calculated between groups of endophytic isolates from the same plant, from different plants and from different geographic origins. These estimates were done in order to evaluate the genetic diversity among the intra- and intergroups according to Nei's equations [18]. The intragroup genetic distance was estimated by the arithmetic mean of the distance between each of the isolates compared in pairs [19]. The intergroups distances were calculated for groups of different plants and different geographic origins as the arithmetic mean of all the distances between the two analyzed groups [19]. These values were calculated with Kimura-2-Parameter [20] with the software MEGA (version 3.1) [21].

### 2.6. Nucleotide and Haplotype Diversity

Average pairwise differences were estimated from comparisons within a library of the number of sequence differences between a given clone and all other clones [22] (Table 3). To estimate genetic diversity within the two libraries, some indexes were calculated using the distance method with Kimura-2-parameter substitution nucleotide model. Average pairwise differences and nucleotide diversity were calculated for each library, as well as molecular indexes, such as number of gene copies and haplotypes, total number of loci, usable loci, polymorphic sites, and gene diversity for each data set. Nucleotide diversity was estimated from the number of variable positions for aligned sequences in a given library.

### 2.7. Genetic Differentiation (*F*_ST_) and Gene Flow (Nm)

*F*_ST_ values were used to evaluate the genetic diversity within the groups of isolates in relation to the total genetic diversity according to the equation *F*_ST_ = (*θ*_*T*_ − *θ*_*W*_)/*θ*_*T*_, where *θ*_*T*_ is the genetic diversity of all isolates and *θ*_*W*_ is the diversity within the group of isolates [23]. Analysis of molecular variance (AMOVA) was performed using Arlequin version 3.0 [24]. Population structures were defined on the basis of the phylogenetic clusters we obtained. A hierarchical analysis of variance was carried out to partition the total variance into variance components attributable to interindividual and/or interpopulation differences. Variance components were then used to compute fixation indices, and their significance was tested at 1000 permutations, as described by Excoffier et al. [22]. Gene flow was calculated by the number of migrants per generation (Nm) according to equation 4 from Hudson et al. [25] using the software DNAsp version 4.50.3 [12].

### 2.8. Genetic Relationships

The aligned sequences were used to verify the genetic relationships among the isolates from same and different plant of Tahiti acid lime from the two places. Dendrograms were built using the Distance method, the grouping algorithm Neighbor Joining [26] nucleotide substitution model Kimura-2-parameter [20] with the software MEGA (version 3.1) [21]. Method reliability was calculated by bootstrapping [27] with 1,000 repetitions by the same software. Dendrograms were built to observe the similarity within the groups of isolates and with the *Guignardia* DNA sequences from different species obtained from GenBank (site http://www.ncbi.nlm.nih.gov/). The *Guignardia* ITS1-5.8S-ITS2 DNA sequences included in each analysis file were *G. citricarpa clone 75* (ID:AF346782.1); *G. citricarpa* (ID: AF346772.1); *G. mangiferae voucher* ICMP 8336 (ID:AY816311.1); *G. mangiferae* (ID: AM403717.1); *G. laricina *(ID:AB041245.1); *G. philoprina *(ID:AB095507.1); *G. philoprina specimen-voucher* CBS 447.68 (ID: AF312014); *G. aesculi* (ID:AB095504.1); *G. vaccinii* (ID:AB041244.1); *G. bidwellii* (ID:AB095511.1); *G. bidwellii* (ID: AB095505); *G. bidwellii* (ID: AB095509); *G. gaultheriae* (ID: AB095506.1); *G. gaultheriae* (ID: AB095506); *Phyllosticta pyrolae* (ID: AF312010); *Phyllosticta pyrolae* (ID: AB041242) e *Phyllosticta spinarum* (ID: AF312009). 

### 2.9. Pathogenicity Tests

Pathogenicity tests were performed according Baldassari et al. [13] using 22 isolates from Estiva Gerbi/Conchal/SP (3 isolates from VC group, 2 isolates from IV group, 4 isolates from NC group, 2 from IN group, 3 from PC group, 3 from IP group, 3 from FE group, and 2 from IE group). The isolates were inoculated on sweet orange “Pera” in January/February of 2007. Fruits were harvested in September 2007 and evaluated for the presence/absence of classic symptoms of CBS.

## 3. Results

### 3.1. Sampling

*Guignardia* typical colonies were obtained from all the samples of asymptomatic leaves, in the same and in different plants and geographic origins, in a total of 208 isolates. The samples, from the same and from different plants in the two different regions, were composed of a minimum of 24 isolates and a maximum of 26 isolates (Table 1).

### 3.2. Culture Characterization of *Guignardia* sp. in Oatmeal (OA) Media

**Figure 1:**
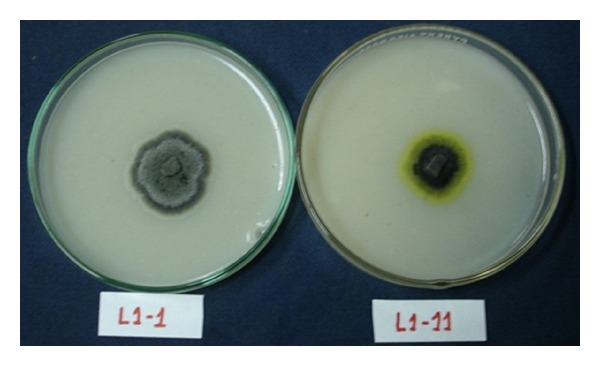
Isolates of *G. mangiferae *(right) and* G. citricarpa *(left) obtained from the same plant of “Tahiti” acid lime in Estiva Gerbi/SP. The two species coexist in this citrus species, but “Tahiti” acid lime does not show symptoms CBS sympthoms.

All 208 *Guignardia* isolates were submitted to characterization in oatmeal media. Among them, eight presented a yellow halo around the colonies (Figure 1), which is considered indicative of *G. citricarpa* species, which is pathogenic to citrus plants [3, 13]. These eight isolates were not used in our population studies, because they belong to the other species. The other 200 isolates did not present a yellow halo, indicative of nonpathogenic isolates.

### 3.3. Amplification and Sequencing of ITS1-5.8S-ITS2

DNA from the isolates was used to amplify the ITS1-5.8S-ITS region. All isolates showed a characteristic band with approximately 800 bp in agarose gel. When submitted to sequencing, all isolates showed a fragment of around 780 bp in length.

### 3.4. Analysis of Obtained DNA Sequences

The obtained sequences were submitted to a quality analysis in order to use only those that showed high quality. All used sequences showed the desired quality up to 20, according to the software Phred/Phrap/Consed. Sequences were edited and ends trimmed. This was done in order to prevent errors during the posterior analysis. All sequences were submitted to GenBank (http://www.ncbi.nlm.nih.gov/genbank/) and its ID are showed in Supplementar Data.

### 3.5. Intra- and Intergroups Genetic Distances

All isolates showed small genetic distances, indicating high genetic similarity. When all isolates from the same geographic region were analyzed as one single group (intragroup), Itaboraí isolates presented a genetic distance slightly lower (0.036) than the group of isolates from Estiva Gerbi (0.032). The genetic distance between (intergroup) isolates from these two regions was 0.0358.

 When analyzed according to sampling, isolates from Estiva Gerbi obtained from different plants of “Tahiti” (LC) presented the highest intragroup genetic distance (0.02831), whereas the lowest was presented by isolates from the plant L1 (0.02270) (Table 1).

Itaboraí isolates from one plant (P1) of “Tahiti” presented the highest intragroup genetic distance (0.04210), whereas the lowest was found for isolates from different plants (P, 0.02916), probably because the same isolate was sampled in different plants.

The highest intergroup genetic distance was presented by isolates from different geographic origins (Table 2). This was presented by Estiva Gerbi isolates of the plant L3 and isolates from Itaboraí also obtained from one plant (P1, 0.04634). Among all the populations, these two can be considered the ones with the highest genetic divergence.

The lowest divergence was presented by groups of isolates from the same geographic origin, Estiva Gerbi, by the group of isolates from “Tahiti” different plants (LC) and those obtained in same plant (L1, 0.02585). These two populations can be considered the ones with the lowest genetic diversity.

### 3.6. Nucleotide and Haplotype Diversity

The diversity indexes show that the highest genetic diversity was found for the groups of isolates from the same plants, Itaboraí/RJ, P1 and P2 (Table 3). These two groups of isolates showed the highest number of polymorphic sites, mean number of pairwise distances, and nucleotide diversity, with each sequence representing one haplotype for IV group. For these groups, 24 haplotypes were found among the 30 studied isolates. The lowest genetic diversity was found in São Paulo State for the groups of isolates from the plants L3 and L1. These two groups presented the lowest number of polymorphic sites, mean number of pairwise distances, and nucleotide diversity, with L3 group presenting 21 haplotypes among the 30 studied isolates. Analysis also revealed that populations collected from one plant, those collected from different plants from the same region, as well as those from the two geographic regions shared haplotypes (Table 4). Among the Estiva Gerbi populations, L2 and L3 shared, the highest number, 12 haplotypes. Among the Itaboraí populations the highest number was found with population P in relation to P2 and P3, sharing 7 haplotypes.

### 3.7. Genetic Differentiation (*F*_ST_) and Gene Flow (Nm)

According to the F values, little-to-moderate genetic differentiation of *G. mangiferae *populations was observed at the various hierarchical levels (among regions, among populations within regions, and within populations, Table 5). The analysis of the ITS1-5.8S-ITS2 DNA sequence indicated that the genetic differentiation of *G. mangiferae *within each sample was significant (*F*_ST_ = 0.18006, *P* ≤ 0.0001), representing 81.99 percent of the observed genetic diversity. The fixation index among populations within regions was also significant, (*F*_SC_ = 0.15722, *P* ≤ 0.0001), representing 15.42% of the observed genetic diversity. The fixation index among the two regions is almost insignificant (*F*_CT_ = −0.02710, *P* ≤ 0.0001) representing 2.71 of the observed genetic differentiation. This indicates that there is gene flow between regions.

When differentiation indexes were calculated for groups of isolates according to samples from the same or different plants (Table 5), it was observed that in Itaboraí/RJ, the highest differentiation was observed for P2 and P3 (*F*_ST_ = 0.07443, *P* ≤ 0.005). The lowest was observed for P and P1 (*F*_ST_ = −0.01585, *P* ≤ 0.005). In Estiva Gerbi/SP, the highest differentiation was observed for LC and L3 populations (*F*_ST_ = 0.36085, *P* ≤ 0.005), whereas the lowest for LC and L1 (*F*_ST_ = 0.01093, *P* ≤ 0.005). Between the two regions, L3 and P3 showed the highest genetic differentiation (*F*_ST_ = 0.25429, *P* ≤ 0.005), and L1 and P1 presented the lowest (*F*_ST_ = −0.00163, *P* ≤ 0.005, not significant).

 Gene flow was also detected among the studied populations (Table 6). All sampled populations presented gene flow at different levels. The highest level of gene flow between populations from Itaboraí/RJ was detected between populations P and P1 (16.03 migrants per generation) and the lowest between P2 and P3 populations of the same plant (3.11 migrants per generation).

 In Estiva Gerbi/SP, the highest level of gene flow between populations was detected for the sample of different plants of LC and the plant L1 (22.6 migrants per generation), and the lowest between the LC population and L3 population (0.44 migrants per generation). The highest levels of gene flow were shown when populations from the two geographic origins were compared (L1 and P1, 153.6 migrants per generation).

### 3.8. Genetic Relationships

**Figure 2:**
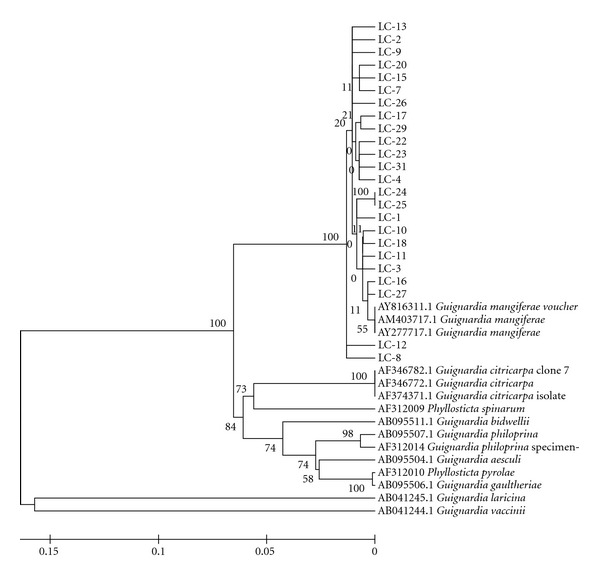
Phylogenetic relationships inferred with DNA sequences from the ITS1-5.8S-ITS2 region of “Tahiti” acid lime isolates obtained from different plants from EstivaGerbi/SP. All isolates showed high similarity with *G. mangiferae *and also one to another. The highest divergence was shown for *G. vaccinii* and *G. laricina*. Tree was obtained by Distance Method using NJ algorithm.

**Figure 3:**
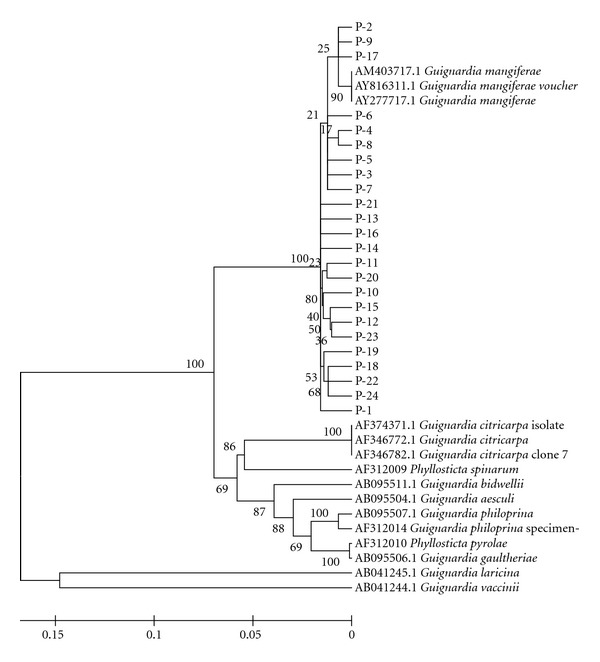
Phylogenetic relationships inferred with DNA sequences from the ITS1-5.8S-ITS2 region of “Tahiti” acid lime isolates obtained from different plants from Itaboraí/RJ. All isolates showed high similarity with *G. mangiferae *and also one to another. Highest divergence was shown to *G. vaccinii* and *G. laricina*. Tree was obtained by Distance Method using NJ algorithm.

**Figure 4:**
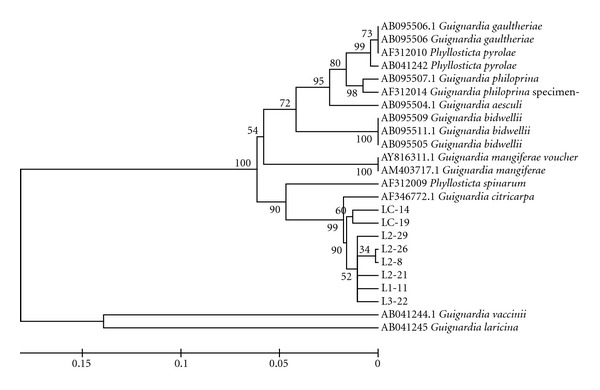
Phylogenetic relationships presented by isolates obtained from “Tahiti” acid lime, showing that this plant is host to *G. citricarpa*, despite not presenting any symptoms of CBS. Tree was obtained by Distance Method using NJ algorithm.

 The six populations were analyzed to verify the similarity of the isolates between the populations sampled and between the samples and the sequences obtained from GenBank. The 200 isolates that did not show a yellow halo in oatmeal media were grouped with *G. mangiferae* sequences obtained from GenBank, showing great similarity among them, as demonstrated by the populations obtained from different plants from Estiva Gerbi (LC, Figure 2) and different plants from Itaboraí (P, Figure 3). The eight isolates that showed a yellow halo were grouped with *G. citricarpa* (Figure 4).

### 3.9. Pathogenicity Tests

**Figure 5:**
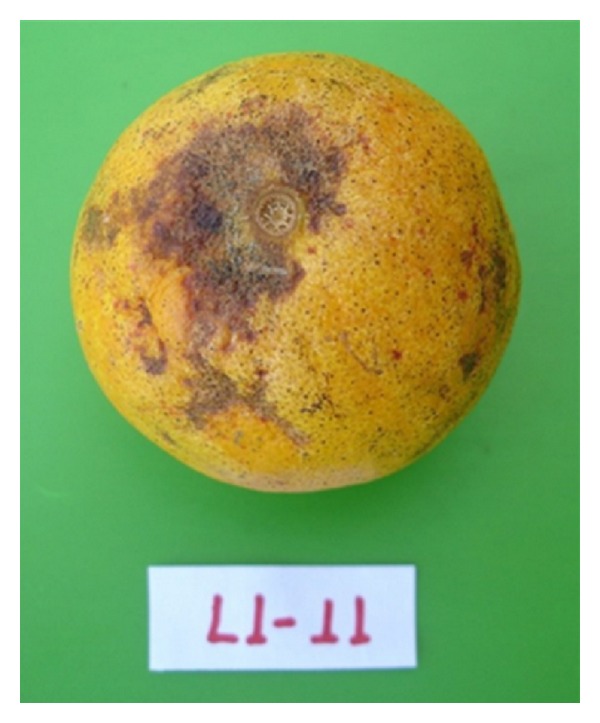
Pathogenicity test with L1-11 isolate showing the typical symptoms of CBS, hard and freckled spot.

Pathogenicity tests using 30 isolates were conducted in order to verify whether they could cause disease in inoculated fruits. None of the 22 isolates that did not show yellow halo in oatmeal media and that were grouped with *G. mangiferae* from GenBank caused any symptoms of CBS in the inoculated fruits. Only the eight isolates that presented a halo in oatmeal media and were grouped with *G. citricarpa* sequences of GenBank caused symptoms in fruits, confirming that they belong to *G. citricarpa* species. The fruits showed mainly freckled and hard spots (as shown in Figure 5).

## 4. Discussion

Because the host plant “Tahiti” acid lime does not show symptoms of the presence of *G. mangiferae*, little is known about the genetic diversity and population indexes of this endophytic organism. We performed a study on the genetic variation and population differentiation of *G. mangiferae*, a fungus that is easily isolated from citrus plants. The ITS ribosomal DNA sequences were found to contain adequate levels of genetic variation to assess population differentiation, and, as far as we know, this is the first report on the population differentiation of *G. mangiferae*. The nuclear rDNA repeat unit is a useful area of the genome to examine for polymorphisms, because of the juxtaposition of conserved and variable regions and its high copy number [28].

Moreover, the ease with which ITS rDNA sequence data can be obtained and the existence of large, phylogenetically referenced databases of ITS rDNA sequences for endophytes underscores the utility of this region for providing a first, if limited, approximation of genotypic differences among samples. ITS rDNA data can obscure species boundaries in some clades, include non-orthologs in some taxa, and exhibit different rates of evolution among different fungal lineages [29].

SNP markers showed that they are useful markers for detecting genetic variation within and between populations of *G. mangiferae*. These markers revealed a moderate-to-low degree of genetic variability within and among the six studied populations. The diversity indexes for the *G. mangiferae* populations in the two geographic areas showed similar results, and AMOVA analysis showed that the most diversity (81.99%) was found within the populations. A moderate diversity was found within regions (15.30%) and can possibly be due to the influences of the host over the populations and to the climatic and soil conditions. Low genetic diversity was found between the populations of Estiva Gerbi/SP and Itaboraí/SP, meaning that the same or similar haplotypes were found in all populations, despite the fact that the two geographical areas are distant from each other (around 800 km). The fact that *G. mangiferae* have several distinct hosts possibly affects the distribution of the diversity within and among populations. We believe that the higher values of genetic differentiation showed by some populations are caused by this. As sampling was done randomly, we probably sampled haplotypes that could be originated from different hosts. Some of the known hosts of *G. mangiferae* are commonly found near citrus orchards, like eucalyptus (*Eucaliptus* sp.), mango (*Mangifera indica*), guava (*Psidium guajava*), and native hosts like jaboticaba (*Myrciaria cauliflora*) and Surinam cherry (*Eugenia uniflora*). Eucalyptus is also commonly used to protect citrus orchards from winds and to avoid the introduction and dissemination of pathogens to the culture.

 This study showed that populations of *G. mangiferae* shared haplotypes and demonstrated gene flow between populations from the same geographic origin and also between the two regions. We believe that this is because the dispersal mechanisms of *G. mangiferae *allied with the colonization of different hosts. Despite the fact that these mechanisms are not well elucidated for *G. mangiferae*, we believe that they are very similar to those presented by *G. citricarpa*. Propagation structures in *G. citricarpa *are either sexually formed ascospores or asexual pycnidia. The fungal spores generated by mitosis “conidia” formed inside specialized organs “pycnidia” are frequent in *G. citricarpa *and are found on fruit lesions during the ripening stage, but they are unlikely to function as dispersal units over long distances [30]. In *G. mangiferae*, we believe that these structures are formed without the presence of symptoms in fruits and leaves. Ascospores, whether formed by a homo- or heterothallic process on fallen leaves, are tiny and may disperse over both relatively short and large distances [31], whereas pycnidia are large and heavy and likely to disperse primarily over short distances [30]. Ascospores of *G. mangiferae* were also detected in fallen leaves [13]. Because of its different hosts, *G. mangiferae* has probably adapted well to conditions during the different seasons, and the generation of dissemination structures is not restricted by some conditions. Different hosts can also permit generation of higher amounts of dissemination structures that can spread over distant regions, facilitating the search for new hosts. Thus, it was demonstrated that populations of *G. mangiferae* present low-to-moderate genetic diversity, but show little-to-moderate levels of population differentiation. As gene flow was detected among the studied populations and they share haplotypes, it is possible that all populations, from citrus and also from the other known hosts of this fungus, belong to one great panmictic population.

“Tahiti” acid lime plants did not show symptoms of CBS, despite the fact that they permit colonization by *G. citricarpa*, a behavior that is also presented by the sour orange (*C. aurantium*) [5]. For “Tahiti” acid lime, this behavior was proposed to be identified like insensible for *G. citricarpa* [13]. These same authors also believe that these two citrus species could have an important role in the epidemiology of *G. citricarpa*, whose extent was unknown at the time.

 On the other hand, *G. mangiferae* is known as a causal agent of fruit and foliar diseases in mango, guava, and banana (*Musa *sp). Despite the fact that the fungi received names according the host (*G. psidium* when isolated from symptomatic fruits and leaves in guava and *G. musae* when isolated from symptoms on banana fruits), recent results showed that all these species probably belong to *G. mangiferae* [5].

 The fact that sometimes the same fungi is identified under different names makes the correct identification of new forms difficult. Despite being identified as *G. mangiferae*, Baayen et al. [3] concluded that these species (whose anamorph is *P. anacardiacearum*) are the same as *G. endophyllicola *(anamorph *P. capitalensis*). In addition, Okane et al. [32] demonstrated that *P. capitalensis* (teleomorph *G. endophyllicola*) exists widely as an endophyte in ericaceous plants. The results obtained by Pandey et al. [28] also support the findings of Okane et al. [32] and Baayen et al. [3], concluding that *P. capitalensis* has a wide host range as an endophytic fungus. Pandey et al. [28] also believe that the wide host range of *P. capitalensis* suggests that this fungus may have been described several times as different species, especially since a species name in *Phyllosticta* is, in many instances, linked to the host.

 The fact that none of the isolates of *G. mangiferae* from this work caused symptoms on sweet orange fruits, as shown by pathogenicity tests, reinforces the hypothesis that *G. mangiferae* is actually endophytic for citrus.

 Despite the fact that the term “endophyte” is employed for all organisms that inhabit plants, mycologists have come to use the term “fungal endophyte” for fungi that inhabit plants without causing visible disease symptoms, and the term refers only to fungi at the moment of detection without regard for the future status of the interaction [33].

 It is known that all fungi invading plant foliage have an asymptomatic period in their life cycle that varies from an imperceptibly short period (like plant pathogens) to a lifetime (like *Neotyphodium* endophytes in grasses) [34].

 Beneficial effects for hosts being colonized by endophytes include increased drought tolerance, deterrence of insect herbivores, protection against nematodes, and resistance against fungal pathogens [35]. The last is also true for endophytes found in some tropical grasses [36]. Antipathogen protection mediated by endophytes has also been observed in different situations. For example, endophytic fungi have been found to protect tomatoes [37] and bananas [38] from nematodes, and beans and barley from fungal pathogens [33]. However, even with the accumulating evidence that endophytic fungi can reduce pathogen damage in grasses and other host plants, little is known about the generality of this role in natural systems and whether it can be exploited as a biocontrol strategy in crop protection [39].

 In the specific case of *G. mangiferae*, there is evidence that the guignardic acid that is produced by this species exhibits potent antimicrobial activity [2].

 Some authors hypothesize that there are no neutral interactions, but rather that endophyte-host interactions involve a balance of antagonisms, irrespective of the plant organ infected [33]. There is always at least a degree of virulence on the part of the fungus enabling infection, whereas defense of the plant host limits the development of fungal invaders and disease. It is also hypothesized that the endophytes, in contrast to known pathogens, generally have far greater phenotypic plasticity and thus more options than pathogens: infection, local but also extensive colonization, latency, virulence, pathogenity, and (or) saprophytism. This phenotypic plasticity is a motor of evolution [33].

 Available evidence suggests that *Botryosphaeriaceae*, which are important endophytics for woody plants, are horizontally transmitted between individuals [40]. These authors also believe that the fact that they are also fairly commonly associated with the seeds of some plants raises the possibility of some level of vertical transmission, but more studies on whether vertical transmission can occur via systematic infection, or via asexual sporulation from a seed infection, followed by infection of the growing plant are needed to provide an important level of understanding regarding the ecological and evolutionary determinants of Botryosphaeriaceae-plant interactions.

 A number of studies suggest that endophytes of woody plants are rather loosely associated with their hosts, with higher correlation between endophyte communities in a specific location, than with a specific host in different locations [34, 41, 42]. This is an also an important question to be addressed in future studies of *G. mangiferae* populations, if populations of different hosts are related one to each other, the range of the presented genetic diversity, and its genetic differentiation. Studies to better evaluate properties like the antifungal substances produced by these fungi, as well as examination of the possibilities of using endophytes for biological control, to replace the actual fungicides are used in Brazilian citriculture.

## Supplementary Material

This Table contains the Guignardia mangiferae isolates used for this study. ITS1-5.8S-ITS2 DNA region of each isolate was amplified in order to perform the population genetic structure described on this research. The DNA sequence was deposited in GenBank and received an Acession Number presented on Table.

## Figures and Tables

**Table 1:** Intragroup genetic distances for isolates from asymptomatic tissues. The genetic distance was estimated by the arithmetic mean of the distance between each of the isolates compared in pairs.

Origin	Groups of isolates	Number of isolates	Intragroup distances
Estiva Gerbi/SP	LC—acid lime “Tahiti” different plants	26	0.02831
	L1—acid lime “Tahiti” same plant	25	0.02270
	L2—acid lime “Tahiti” same plant	25	0.02311
	L3—acid lime “Tahiti” same plant	25	0.02757

Itaboraí/RJ	P—acid lime “Tahiti” different plants	25	0.02916
	P1—acid lime “Tahiti” same plant	24	0.04210
	P2—acid lime “Tahiti” same plant	25	0.03763
	P3—acid lime “Tahiti” same plant	25	0.03344

**Table 2:** Intergroup genetic distances for isolates from asymptomatic tissues. The genetic distances were calculated for groups of different plants and different geographic origins as the arithmetic mean of all the distances between the two analyzed groups.

	LC	L1	L2	L3	P	P1	P2	P3
LC	—							
L1	**0.02585**	—						
L2	0.03325	0.02900	—					
L3	0.04447	0.03997	0.02898	—				
P	0.03078	0.02712	0.02954	0.03915	—			
P1	0.03577	0.03256	0.03654	**0.04634**	0.03499	—		
P2	0.04048	0.03664	0.03460	0.04200	0.03469	0.04227	—	
P3	0.03593	0.03259	0.03222	0.04174	0.03214	0.03908	0.03732	—

**Table 3:** Diversity indexes calculated for 8 populations of *G. mangiferae* from “Tahiti” acid lime originating from two different geographic areas.

Groups of Isolates	Number of gene copies	Number of sequences/haplotypes	Number of polymorphic sites	Mean no of pairwise differences	Nucleotide diversity
LC	24	24	218	47.841 ± 21.313	0.077 ± 0.038
L1	25	24	180	43.558 ± 19.434	0.071 ± 0.035
L2	25	21	134	44.308 ± 19.762	0.072 ± 0.036
L3	25	22	135	40.068 ± 17.902	0.065 ± 0.032
P	25	24	234	50.381 ± 22.427	0.081 ± 0.040
P1	24	24	304	64.735 ± 28.725	0.102 ± 0.050
P2	25	24	263	62.036 ± 27.541	0.099 ± 0.049
P3	25	24	255	57.804 ± 25.684	0.091 ± 0.045

**Table 4:** Number of haplotypes shared between populations sampled in “Tahiti” acid lime orchards.

	LC	L1	L2	L3	P	P1	P2	P3
LC	—							
L1	01	—						
L2	—	—	—					
L3	—	02	12	—				
P	03	07	—	—	—			
P1	07	09	—	—	06	—		
P2	—	—	—	—	07	04	—	
P3	—	—	—	—	07	04	02	—

**Table 5:** AMOVA analysis comparing results of genetic variation from *G. mangiferae *sampled from the same and from different plants of “Tahiti” acid lime in two geographic areas.

Source of variation	d.f.	Sum of squares	Variance components	Percentage of variation	Fixation indices
Among regions	1	272.800	0.85353 Va	2.71	*F*_CT_ = −0.02710 (*P* < 0.0001)
Among populations within regions	6	1022.258	4.81828 Vb	15.30	*F*_SC_ = 0.15722 (*P* < 0.0001)
Within populations	200	5992.067	25.82787 Vc	81.99	*F*_ST_ = 0.18006 (*P* < 0.0001)

Total	207	7287.125	31.49969		

**Table 6:** Genetic differentiation of populations and gene flow (in brackets) obtained for different samples of acid lime “Tahiti.”

	LC	L1	L2	L3	P	P1	P2	P3
LC	—							
L1	0.01093 (22.6)	—						
L2	0.20840 (0.95)	0.18690 (1.09)	—					
L3	**0.36085 (0.44)**	0.33707 (0.49)	0.10986 (2.03)	—				
P	0.06500 (3.6)	0.04406 (5.42)	0.08175 (2.81)	0.24818 (0.76)	—			
P1	0.02040 (12)	**−0.00163 (−153.6)**	0.09212 (2.46)	0.23197 (0.83)	−0.01585 (−16.03)	—		
P2	0.18560 (1.2)	0.17024 (1.22)	0.10056 (2.24)	0.21508 (0.91)	0.04057 (5.91)	0.05040 (4.71)	—	
P3	0.17617 (1.17)	0.15834 (1.33)	0.10396 (2.15)	0.25429 (0.73)	0.04249 (5.63)	0.05023 (4.73)	0.07443 (3.11)	—
