# Supplementary material for: Genetic Diversity and Population Differentiation of Guignardia mangiferae from “Tahiti” Acid Lime
Source: ScientificWorldJournal. 2012 Apr 19;2012:125654. doi: 10.1100/2012/125654 (PMC3348534; doi:10.1100/2012/125654)
Supplement: Supplementary file 1 — This Table contains the Guignardia mangiferae isolates used for this study. ITS1-5.8S-ITS2 DNA region of each isolate was amplified in order to perform the population genetic structure described on this research. The DNA sequence was deposited in GenBank and received an Acession Number presented on Table. [file 125654.f1.doc]

**SUPPLEMEN**TAR DATA

| **Isolate//GenBank ID** | **Isolate//GenBank ID** | **Isolate//GenBank ID** | **Isolate//GenBank ID** | **Isolate//GenBank ID** | **Isolate//GenBank ID** | **Isolate//GenBank ID** | **Isolate//GenBank ID** |
| --- | --- | --- | --- | --- | --- | --- | --- |
| P-1 // JQ317397 | P1-1 // JQ317421 | P2-1 // JQ317455 | P3-1 // JQ317479 | LC-1 // FJ769691 | L1-1 // JQ317503 | L2-1 // JQ317551 | L3-1 // JQ317575 |
| P-2 // JQ317398 | P1-2 // JQ317422 | P2-2 // JQ317461 | P3-2 // JQ317485 | LC-2 // FJ769699 | L1-2 // JQ317511 | L2-2 // JQ317557 | L3-2 // JQ317581 |
| P-3 // JQ317399 | P1-3 // JQ317423 | P2-3 // JQ317462 | P3-3 // JQ317486 | LC-3 // FJ769701 | L1-3 // JQ317512 | L2-3 // JQ317558 | L3-3 // JQ317582 |
| P-4 // JQ317400 | P1-4 // JQ317424 | P2-4 // JQ317463 | P3-4 // JQ317487 | LC-4 // FJ769702 | L1-4 // JQ317513 | L2-4 // JQ317559 | L3-4 // JQ317583 |
| P-5 // JQ317401 | P1-5 // JQ317425 | P2-5 // JQ317464 | P3-5 // JQ317488 | LC-6 // FJ769635 | L1-5 // JQ317497 | L2-5 // JQ317560 | L3-5 // JQ317584 |
| P-6 // JQ317402 | P1-6 // JQ317426 | P2-6 // JQ317465 | P3-6 // JQ317489 | LC-7 // FJ769703 | L1-6 // JQ317514 | L2-6 // JQ317561 | L3-6 // JQ317585 |
| P-7 // JQ317403 | P1-7 // JQ317427 | P2-7 // JQ317466 | P3-7 // JQ317490 | LC-8 // FJ769704 | L1-7 // JQ317496 | L2-7 // JQ317562 | L3-7 // JQ317586 |
| P-8 // JQ317404 | P1-8 // JQ317428 | P2-8 // JQ317467 | P3-8 // JQ317491 | LC-9 // FJ769705 | L1-8 // JQ317515 | L2-9 // JQ317564 | L3-8 // JQ317587 |
| P-9 // JQ317405 | P1-9 // JQ317429 | P2-9 // JQ317468 | P3-9 // JQ317492 | LC-10 // FJ769683 | L1-9 // JQ317516 | L2-10 // JQ317541 | L3-9 // JQ317588 |
| P-10 // JQ317406 | P1-10 // JQ317430 | P2-10 // JQ317445 | P3-10 // JQ317469 | LC-11 // FJ769684 | L1-10 // JQ317498 | L2-11 // JQ317542 | L3-10 // JQ317565 |
| P-11//JQ317407 | P1-11 // JQ317431 | P2-11 // JQ317446 | P3-11 // JQ317470 | LC-12 // FJ769685 | L1-12 // JQ317499 | L2-12 // JQ317543 | L3-11 // JQ317566 |
| P-12 // JQ317408 | P1-12 // JQ317432 | P2-12 // JQ317447 | P3-12 // JQ317471 | LC-13 // FJ769686 | L1-13 // JQ317500 | L2-13 // JQ317544 | L3-12 // JQ317567 |
| P-13 // JQ317409 | P1-13 // JQ317433 | P2-13 // JQ317448 | P3-13 // JQ317472 | LC-15 // FJ769687 | L1-14 // JQ317501 | L2-14 // JQ317545 | L3-13 // JQ317568 |
| P-14 // JQ317410 | P1-14 // JQ317434 | P2-14 // JQ317449 | P3-14 // JQ317473 | LC-16 // FJ769688 | L1-16 // JQ317502 | L2-15 // JQ317546 | L3-14 // JQ317569 |
| P-15 // JQ317411 | P1-15 // JQ317435 | P2-15 // JQ317450 | P3-15 // JQ317474 | LC-17 // FJ769689 | L1-18 // JQ317493 | L2-16 // JQ317547 | L3-15 // JQ317570 |
| P-16 // JQ317412 | P1-16 // JQ317436 | P2-16 // JQ317451 | P3-16 // JQ317475 | LC-18 // FJ769690 | L1-20 // JQ317504 | L2-17 // JQ317548 | L3-16 // JQ317571 |
| P-17 // JQ317413 | P1-17 // JQ317437 | P2-17 // JQ317452 | P3-17 // JQ317476 | LC-20 // FJ769692 | L1-21 // JQ317505 | L2-18 // JQ317549 | L3-17 // JQ317572 |
| P-18 // JQ317414 | P1-18 // JQ317438 | P2-18 // JQ317453 | P3-18 // JQ317477 | LC-22 // FJ769693 | L1-23 // JQ317506 | L2-19 // JQ317550 | L3-18 // JQ317573 |
| P-19 // JQ317415 | P1-19 // JQ317439 | P2-19 // JQ317454 | P3-19 // JQ317478 | LC-23 // FJ769694 | L1-24 // JQ317507 | L2-20 // JQ317552 | L3-19 // JQ317574 |
| P-20 // JQ317416 | P1-20 // JQ317440 | P2-20 // JQ317456 | P3-20 // JQ317480 | LC-25 // FJ769695 | L1-25 // JQ317494 | L2-22 // JQ317554 | L3-20 // JQ317576 |
| P-21 // JQ317417 | P1-21 // JQ317441 | P2-21 // JQ317457 | P3-21 // JQ317481 | LC-26 // FJ769696 | L1-26 // JQ317508 | L2-23 // JQ317555 | L3-21 // JQ317577 |
| P-22 // JQ317418 | P1-22 // JQ317442 | P2-22 // JQ317458 | P3-22 // JQ317482 | LC-27 // FJ769697 | L1-27 // JQ317509 | L2-24 // JQ317556 | L3-25 // JQ317578 |
| P-23 // JQ317419 | P1-23 // JQ317443 | P2-23 // JQ317459 | P3-23 // JQ317483 | LC-29 // FJ769698 | L1-28 // JQ317495 | L2-25 // JQ317563 | L3-23 // JQ317579 |
| P-24 // JQ317420 | P1-24 // JQ317444 | P2-24 // JQ317460 | P3-24 // JQ317484 | LC-31 // FJ769700 | L1-29 // JQ317510 | L2-27 // JQ317553 | L3-24 // JQ317580 |
